# Prevalence of metabolic syndrome in a rural area of Brazil

**DOI:** 10.1590/S1516-31802007000300006

**Published:** 2007-05-03

**Authors:** Gustavo Velásquez-Meléndez, Andrea Gazzinelli, Rodrigo Côrrea-Oliveira, Adriano Marçal Pimenta, Gilberto Kac

**Keywords:** Metabolic syndrome X, Hypertension, Obesity, Body mass index, Rural population, Brazil, Síndrome X metabólica, Hipertensão, Obesidade, Índice de massa corporal, População rural, Brasil

## Abstract

**CONTEXT AND OBJECTIVE:**

Metabolic syndrome (MS) is recognized worldwide as an important public health concern. However, little information is available for rural populations in Brazil. The aim was to determine the prevalence and risk factors associated with MS in a rural village in Brazil in 2004.

**DESIGN AND SETTING:**

Cross-sectional population-based study, in Virgem das Graças, a rural community in the Jequitinhonha Valley, State of Minas Gerais.

**METHODS:**

MS was the dependent variable, defined as any three of these risk factors: arterial hypertension, high glucose or triglyceride concentrations, low high-density lipoprotein cholesterol and abdominal obesity. MS prevalence, according to selected socioeconomic and demographic variables (age, skin color, marital status, schooling and smoking habits), was determined in 251 subjects aged 20-88 years. Multivariate logistic regression was used to estimate odds ratios (OR) and 95% confidence intervals.

**RESULTS:**

MS prevalence was 21.6% (7.7% for men and 33.6% for women); the age-adjusted prevalence was 19.0%. The highest prevalences were observed for women > 60 years of age (52.9%) and women with body mass index (BMI) ≥ 25 kg/m^2^ (64%). Age, sex and BMI were associated risk factors for MS, while skin color was only significantly associated with MS for women. The models were adjusted for age, smoking habits, marital status, skin color and schooling.

**CONCLUSIONS:**

BMI and age were independently associated factors for MS in this rural community. These findings provide important evidence on the prevalence of MS as a public health problem, particularly for women and overweight individuals.

## RESUMO

**OBJETIVO E CONTEXTO:**

A síndrome metabólica (SM) é reconhecida mundialmente como importante problema de saúde pública e tem apresentado prevalência crescente. Entretanto, existe pouca informação sobre prevalência da SM em populações rurais brasileiras. Este estudo objetivou determinar a prevalência e os fatores associados a SM em uma comunidade rural do Brasil, em 2004.

**TIPO DE ESTUDO E LOCAL:**

Estudo transversal de base populacional, conduzido em Virgem das Graças, comunidade rural localizada no Vale do Jequitinhonha, Estado de Minas Gerais, Brasil, 2004.

**MÉTODOS:**

SM foi definida pelo critério do National Cholesterol Education Program Adult Treatment Panel (NCEP/ATP III) como a co-ocorrência de pelo menos três dos seguintes fatores: hipertensão arterial, altos níveis séricos de glicose ou triglicérides, baixos níveis da lipoproteína de alta densidade, e obesidade abdominal. A prevalência de SM foi calculada segundo variáveis socioeconômicas e demográficas em 251 pessoas (117 homens, 134 mulheres) com idades entre 20-88 anos. Utilizou-se regressão logística para estimar *odds ratio* (OR) e seus intervalos de confiança de 95%.

**RESULTADOS:**

Prevalência da SM foi 21,6% (7,7% para os homens e 33,6% para as mulheres). A prevalência global ajustada por idade foi 19,0%. Prevalências altas foram encontradas em mulheres > 60 anos (52,9 %) e em mulheres com Índice de Massa Corporal (IMC) ≥ 25 (64%). A análise ajustada por hábito de fumar, status marital, cor de pele e escolaridade revelou que o sobrepeso e sexo feminino e a idade como principais fatores de risco relacionado à SM.

**CONCLUSÕES:**

A síndrome metabólica representa um potencial problema de saúde pública particularmente em mulheres e indivíduos com sobrepeso na área rural estudada. O sobrepeso, a idade acima de 60 anos e o sexo feminino foram considerados fatores independendentes associados a essa condição.

## INTRODUCTION

Chronic and degenerative diseases are the most important causes of mortality among the population of Brazil. In 2001, diseases of the circulatory system such as heart ischemia and cerebrovascular disorders were the primary causes of death, responsible for approximately 32% of the cases, followed by external trauma (15%) and neoplasia (14%). In contrast, infectious and parasitic diseases were responsible for just 6% of deaths during the same period.^1^

Overall, in the past decades, the Brazilian population has experienced relatively rapid socioeconomic improvement, resulting in many lifestyle modifications that have promoted increased prevalence of obesity and associated diseases, such as diabetes and dyslipidemias, which are considered to be part of the nutritional transition process. For example, consumption of high energy density foods that are rich in fats and sugar has increased. In contrast, physical activity and grain/legume intake have decreased.^2^

The main risk factors for the occurrence of circulatory diseases are obesity, hypertension, diabetes, metabolic dyslipidemias and an inadequate lifestyle. Metabolic syndrome (MS) has been described as being the condition in which at least three of the acknow-ledged risk factors are observed simultaneously.^3^ Epidemiological studies have shown that MS is relatively common in societies that have undergone alterations in lifestyle habits, typically occasioned by economic and technological changes. Thus, it has been estimated that 44% of the American population above 50 years of age are affected with MS.^4^

Cross-sectional population-based studies have shown that the presence of MS is associated with the risk of myocardial infarction and stroke.^5,6^ These results have been confirmed by cohort studies.^7^ Furthermore, the components of MS, either independently or together, are considered to be important risk factors for mortality, even in the absence of evident diabetes and cardiovascular diseases.^8^ Thus, these studies suggest that diagnosing MS may contribute towards identifying groups that are at increased risk of suffering from cardiovascular diseases and their consequences.

A number of authors have reported high MS prevalence and increased mortality rates in specific populations and ethnic groups, mostly among those that are in the active stages of epidemiological transition and are more vulnerable to cardiovascular diseases.^9–11^ However, the data concerning the prevalence of MS among urban and rural populations in Brazil are very limited.

## OBJECTIVE

The aim of the present study was to determine the prevalence and risk factors associated with MS in a rural area in the state of Minas Gerais (MG), Brazil.

## METHODS

### Area of study and subjects

A cross-sectional population-based study was conducted during 2004 in five settlements, each located between 1 and 5 km from Virgem das Graças, a rural village in the municipality of Ponto dos Volantes, situated in a semiarid region of the Jequitinhonha Valley in the state of Minas Gerais, Brazil. This is one of the rural areas in Brazil whose inhabitants depend primarily on subsistence farming, principally cassava (manioc), maize and rice, together with the raising of livestock. Most of the population are small landowners, while others have their own small, commercial businesses, and still others are migrant workers from other areas in search of work. In spite of access to a public school and a health district outpost, access to health and education is limited.

The settlements studied, referred to as Taboca, Cardoso I, II and III and Sussuarana, are located along the Cardoso and Sussuarana streams, which are both tributaries emptying into the Jequitinhonha River. None of the village communities have local installations that treat their main water supply, nor do they treat their human waste. The Taboca community's main source of water supply is derived from a protected spring that is used as a central community reservoir that supplies water to the village homes. Since the main water source does not produce sufficient quantity of water for the villagers’ daily domestic needs, many use the nearby stream to satisfy their additional needs. On the other hand, most houses in the villages have their own private water supply that is either piped from protected springs or is fetched by hand from streams, canals, unprotected springs or wells. A unique identification number was allocated to each house and to every one of its occupants in all of the villages. At the time of this study, Virgem das Graças had a total population of 685 residents; 278 of them were less than 18 years of age, seven pregnant women, four individuals presented with medical-surgical impediments and 11 emigrants met the exclusion criteria, leaving 385 eligible subjects. Additionally, 134 individuals either did not have any biochemical analysis (n = 34), refused to participate (n = 56) or were absent at the time of the survey (n = 44), and thus were excluded, resulting in a final sample of 251 subjects (117 men and 134 women).

### Data collection

The participants completed a survey questionnaire covering various aspects of their social and economic conditions. The following variables were investigated: age categories (18-29, 30-42, 43-59, 60-88 years), skin color (white, mixed/black), marital status (married, living with partner, single, widowed/separated), smoking habits (smoker, non-smoker, former smoker) and schooling (zero, 1-14 years). In addition, female subjects were asked about their obstetric history (parity).

At the conclusion of the interview, a clinical evaluation of subjects was performed which included anthropometry and a blood test to determine glucose and lipid concentrations.

### Anthropometry

All measurements were carried out in triplicate, by well-trained anthropometrists in accordance with standard procedures,^12^ and the mean values were recorded. Body weight was determined with subjects wearing light clothes and no shoes or socks, using an electronic scale (Filizola^®^ model PL150, São Paulo, Brazil). Height was determined using a wall-mounted, non-extendable two-meter measuring tape (accurate to 0.1 cm) with subjects in a standing position, feet together and head placed according to the Frankfürt plan. Waist circumference (WC) was measured to the nearest millimeter using a non-extendable measuring tape, and taken exactly halfway between the margin of the lowest rib and the iliac crest with participants in a standing position. Body mass index (BMI) was calculated using the expression:


$$\text{BMI}=\text{weight}\left(\text{kg}\right)/{\text{height}}^{2}\;\left(\text{m}\right).$$

### Definition of metabolic syndrome

The definition of MS used in this study was the one recommended by the National Cholesterol Education Program (NCEP),^13^ which is based on the detection of three or more of the following conditions: arterial hypertension (systolic pressure ^≥^ 130 mmHg or diastolic pressure ^≥^ 85 mmHg), high glucose (^≥^ 110 mg/dl), hypertriglyceridemia (TG ^≥^ 150 mg/dl), low level of high-density lipoprotein cholesterol (HDL-C; < 40 mg/dl for males and < 50 mg/dl for females), and abdominal obesity (waist circumference, WC ^≥^ 102 cm for males and ^≥^ 88 cm for females). In this study, individuals undergoing treatment for hypertension or diabetes were automatically classified as presenting those conditions.

### Determination of arterial blood pressure

Arterial blood pressure was determined by an indirect method, using a calibrated mercury sphygmomanometer, following the recommendations of the Joint National Committee^14^ and in accordance with Brazilian Guidelines for Arterial Hypertension.^15^ Measurements were obtained with the subject in a sitting position and the right arm extended at shoulder level: the beginning of the audio indication of auscultation was the marker for recording arterial systolic pressure, and the termination of this indication was the marker for recording the arterial diastolic pressure. Measurements were carried out in triplicate for each subject with an interval of two minutes between readings, and mean values were calculated.

### Biochemical analyses

Blood samples (5 ml) were collected from each subject by venous puncture following a fasting period of 12 h. Aliquots of serum and plasma were obtained by centrifugation of each sample, and were appropriately treated and stored in vials maintained at 4° C until required by the laboratory for biochemical analysis. Colorimetric enzymatic methods were used for determining glucose, triglycerides (TG) and total cholesterol using a Roche Cobas Mira Plus analyzer. The HDL-C concentration was also determined by colorimetric enzymatic assay following precipitation of the low-density lipoprotein cholesterol (LDL-C) and very low-density lipoprotein cholesterol (VLDL-C) fractions using phosphotungstic acid and magnesium chloride. The LDL-C concentrations were calculated by applying the Friedewald equation.^16^

### Statistical analyses

The information collected was entered into a data bank and analyzed using the Epi-Info version 3.3.2 and Statistical Package for the Social Sciences (SPSS) version 8.0 software. MS was defined as the dependent variable, while sex, age, skin color, marital status, smoking habits, schooling, presentation of overweight (BMI ≥ 25 kg/m^2^) and obesity (BMI ≥ 30 kg/m^2^), and (for women) parity, were considered to be the covariables of interest. By applying the χ^2^ test, covariable distributions were initially stratified according to sex, and the prevalences of MS components between the sexes were compared. The statistical significance level was established at 5% (p < 0.05).

Associations between the dependent variable and covariables were determined using bivariate analysis. Odds ratios (OR) and the respective 95% confidence intervals (95% CI) were determined separately for males and females. The final non-conditional multivariate logistic regression model was established using a forward stepwise procedure, separately for females and the whole population. The order of inclusion of the variables in the final model was from the highest to the lowest OR value. The evaluation of these models was performed according to the goodness-of-fit test.

### Ethics committee approval

This study was approved by the Research Ethics Committee of Universidade Federal de Minas Gerais, in accordance with National Health Council Resolution 196/96. All of the subjects who took part in the study were informed about the objectives of the research and their rights as participants, and then were asked to sign a consent form.

## RESULTS

The overall prevalence of MS among the study population was 21.6% (95% CI: 16.6 – 27.1), and it was 33.6% for women and 7.7% for men. When the values were adjusted to take into account the age distribution of the study population in comparison with a standard population of a rural area in Minas Gerais,^17^ the estimated frequency of MS was 19.0% (95% CI: 13.7 – 24.3) for the whole study population, and it was 30.8% for women and 5.8% for men.

Table 1 shows the characteristics of the study population according to sex. Among the variables that presented statistically significant differences between male and female subjects were marital status, smoking habits, overweight and obesity. The frequency of single adults was higher among men, while the frequency of widowed and separated status was higher among women. Smoking was more common among men, while overweight and obesity showed a higher prevalence among women.

**Table 1.** Characteristics of the study population in Virgem das Gra$as, Brazil, according to sex

Characteristics	Males n (%)	Females n (%)	Total	p value*
**Age (years)**				
18-29	25 (21.4)	36 (26.9)	61 (24.3)	
30-42	26 (22.2)	32 (23.9)	58 (23.1)	
43-59	38 (32.5)	32 (23.9)	70 (27.9)	
60-88	28 (23.9)	34 (25.4)	62 (24.7)	0.4646
**Skin color**				
White	42 (35.9)	61 (45.5)	103 (41.0)	
Black/mixed	75 (64.1)	73 (54.5)	148 (59.0)	0.1227
**Marital status**				
Married	66 (56.4)	78 (58.2)	144 (57.4)	
Living with partner	21 (17.9)	17 (12.7)	38 (15.1)	
Single	26 (22.2)	21 (15.7)	47 (18.7)	
Widowed/separated	4 (3.4)	18 (13.4)	22 (8.8)	**< 0.0207**
**Smoking habits**				
Smoker	40 (34.2)	13 (9.7)	53 (21.1)	
Non-smoker	42 (35.9)	110 (82.1)	152 (60.6)	
Former smoker	35 (29.9)	11 (8.2)	46 (18.3)	**< 0.0001**
**Schooling (years)**				
0	42 (35.9)	52 (38.8)	94 (37.5)	
1-14	75 (64.1)	82 (61.2)	157 (62.5)	0.6355
**BMI (**≥ **25 kg/m^2^)**				
Yes	14 (12.0)	50 (37.3)	64 (25.5)	
No	103 (88.0)	84 (62.7)	187 (74.5)	**< 0.0001**
**BMI (**≥ **30 kg/m^2^)**				
Yes	2 (1.7)	15 (11.2)	17 (6.8)	
No	115 (98.3)	119 (88.8)	234 (93.2)	**< 0.0001**
**Parity (number)**				
1-2		26 (22.8)†		
3-4		29 (25.4)		
≥ 5		59 (51.8)		

* *χ^2^ test for proportions;*

† *Twenty of these subjects were primiparae. BMI = body mass index.*

Examination of the individual components that constituted MS in males and females showed that there was a significant difference with regard to HDL-C and waist circumference (Table 2). The prevalence of low HDL-C and abdominal obesity was higher among women than among men. Overall, the number of subjects suffering from arterial hypertension was 62.5%.

**Table 2.** Components of metabolic syndrome among the study population in Virgem das Graças, Brazil, according to sex

Components of metabolic syndrome*	Males	Females n (%)	Total	p value†
**Arterial hypertension**	**n** (%)			
Yes	77 (65.8)	80 (59.7)	157 (62.5)	
No	40 (34.2)	54 (40.3)	94 (37.5)	0.3193
**Diabetes**				
Yes	6 (5.1)	9 (6.7)	15 (6.0)	
No	111 (94.9)	125 (93.3)	236 (94.0)	0.5971
**Hypertriglyceridemia**				
Yes	24 (20.5)	32 (23.9)	56 (22.3)	
No	93 (79.5)	102 (76.1)	195 (77.7)	0.5234
**Low HDL-C**				
Yes	27 (23.1)	66 (49.3)	93 (37.1)	
No	90 (76.9)	68 (50.7)	158 (62.9)	< 0.0001
**Abdominal obesity**				
Yes	5 (4.3)	62 (46.3)	67 (26.7)	
No	112 (95.7)	72 (53.7)	184 (73.3)	< 0.0001
**Number of co-occurring components**				
0	26 (22.2)	27 (20.1)	53 (21.1)	
1	56 (47.9)	33 (24.6)	89 (35.5)	
2	26 (22.2)	29 (21.6)	55 (21.9)	
3	5 (4.3)	25 (18.7)	30 (12.0)	
4	4 (3.4)	17 (12.7)	21 (8.4)	
5	0 (0.0)	3 (2.2)	3 (1.2)	< 0.0001

* *Components defined by the National Cholesterol Education Program^9^ as: arterial hypertension (systolic pressure ≥ 130 mmHg or diastolic pressure ≥ 85 mmHg); glucose after fasting (≥ 110 mg/dl); hypertriglyceridemia (TG ≥ 150 mg/dl); low high-density lipoprotein-cholesterol (HDL-C; males < 40 mg/dl and females < 50 mg/dl); and abdominal obesity (waist circumference; males ≥ 102 cm and females ≥ 88 cm).*

† *χ2 test for proportions.*

Table 3 shows the distribution of MS between males and females in the study population according to the selected covariables of age, skin color, marital status, smoking habits, schooling, BMI and (for women) parity. The prevalence of MS increased with age for both males and females, although the highest frequency (52.9%) was observed in women between the ages of 60 and 88 years. MS was more prevalent among black/mixed women (42.5%) than among white women. Lower prevalence of MS was found in single adults than in other categories, and in subjects with at least one year of schooling in comparison with illiterate subjects.

**Table 3.** Prevalence of metabolic syndrome among the male, female and total study populations in Virgem das Graças, Brazil

Variables	Males		Females		Total	
	n (prevalence)	OR (95% CI)	n (prevalence)	OR (95% CI)	n (prevalence)	OR (95% CI)
**Age (years)**						
18-29	0 (0.0)	1.00	4 (11.1)	1.00	4 (6.6)	1.00
30-42	0 (0.0)	inexact	11 (34.4)	1.87 [0.70 - 5.00]	11 (19.0)	1.71 [0.80 - 3.64]
43-59	5 (13.2)	inexact	12 (37.5)	2.14 [0.79 - 5.79]	17 (24.3)	2.35 [1.01 - 5.43]
60-88	4 (14.3)	inexact	18 (52.9)	9.00 [2.60 - 31.05]	22 (35.5)	7.83 [2.50 - 24.48]
**Skin color**						
White	5 (11.9)	1.00	14 (23.0)	1.00	19 (18.4)	1.00
Mixed/black	4 (5.3)	0.42 [0.10 - 1.64]	31 (42.5)	2.48 [1.16 - 5.28]	35 (23.6)	1.37 [0.73 - 2.56]
**Marital Status**						
Married	6 (9.1)	3.16 [0.21 - 46.72]	29 (37.2)	1.52 [0.37 - 6.25]	35 (24.3)	2.53 [0.76 - 8.36]
Living with partner	2 (9.5)	3.33 [0.30 - 37.25]	5 (29.4)	1.07 [0.37 - 3.08]	7 (18.4)	1.77 [0.69 - 4.60]
Single	0 (0.0)	1.00	4 (19.0)	1.00	4 (8.5)	1.00
Widowed/separated	1 (25.0)	inexact	7 (38.9)	2.70 [0.63 - 11.45]	8 (36.4)	6.14 [1.60 - 23.53]
**Smoking habits**						
Smoker	2 (5.0)	2.15 [0.18 - 24.75]	3 (23.1)	0.54 [0.14 - 2.10]	5 (9.4)	0.42 [0.13 - 1.39]
Non-smoker	1 (2.4)	1.00	39 (35.5)	1.00	40 (26.3)	1.00
Former smoker	6 (17.1)	0.25 [0.05 - 1.35]	3 (27.3)	0.80 [0.12 - 5.10]	9 (19.6)	0.29 [0.10 - 0.78]
**Schooling (years)**						
0	5 (11.9)	2.40 [0.60 - 9.47]	23 (44.2)	2.16 [1.03 - 4.50]	28 (29.8)	2.14 [1.16 - 3.93]
1-14	4 (5.3)	1.00	22 (26.8)	1.00	26 (16.6)	1.00
**BMI (≥ 25 kg/m^2^)**						
Yes	7 (50.0)	inexact	32 (64.0)	9.70 [4.24 - 22.18]	39 (60.9)	17.88 [8.63- 37.05]
No	2 (1.9)	1.00	13 (15.5)	1.00	15 (8.0)	1.00
**BMI (≥ 30 kg/m^2^**)						
Yes	2 (100.0)	50.50 [8.79 - 290.0]	12 (80.0)	10.42 [2.76 - 39.30]	14 (82.4)	22.62 [6.21 - 82.41]
No	7 (6.1)	1.00	33 (27.7)	1.00	40 (17.1)	1.00
**Parity (number)**						
1-2			9 (19.6)	1.00		
3-4			8 (27.6)	2.37 [0.90 - 6.20]		
≥ 5			28 (47.5)	3.71 [1.52 - 9.04]		

*OR = odds ratio; 95% CI = 95% confidence interval; BMI = body mass index.*

The prevalence of MS was significantly higher in overweight (OR = 17.88) and obese (OR = 22.62) subjects for the whole population, while the respective values of OR for MS in overweight and obese women were 9.70 and 10.42. Other factors that influenced the occurrence of MS among women were: ages 60 to 88 years (OR = 9.00), parity ≥ 5 (OR = 3.71), mixed/black skin color (OR = 2.48) and lack of schooling (OR = 2.16). The values for men were not considered sufficiently accurate for analysis (Table 3).

Table 4 shows the results from the final regression analysis model determined for female subjects, and Table 5, for the population as a whole. The variables that remained statistically associated with MS in the final model were overweight and age, while skin color was only significantly associated with MS in females.

**Table 4.** Adjusted values for odds ratios and 95% confidence intervals relating to regression analysis models for metabolic syndrome determined for the female study population in Virgem das Graças, Brazil

Model	OR (95% CI)
**Model 1. BMI**	
BMI (≥ 25 kg/m^2^)	9.70 [4.24-22.18]
**Model 2. BMI + age**	
BMI (≥ 25 kg/m^2^)	11.03 [4.37-27.85]
Age (years)	
(30-42)	2.80 [0.86-9.13]
(43-59)	3.69 [1.11-12.37]
(60-88)	10.59 [2.60-43.22]
**Model 3. BMI + age + parity**	
BMI (≥ 25 kg/m^2^)	11.50 [4.47-29.57]
Age (years)	
(30-42)	2.63 [0.80-8.71]
(43-59)	2.98 [0.75-11.91]
(60-88)	7.10 [1.31-38.50]
Parity (living with partner)	
(3-4)	1.87 [0.51-6.90]
(≥ 5)	1.06 [0.29-3.92]
**Model 4. BMI + age + skin color**	
BMI (≥ 25 kg/m^2^)	11.08 [4.31-28.46]
Age (years)	
(30-42)	3.06 [0.92-10.20]
(43-59)	3.35 [0.99-11.31]
(60-88)	11,4 [2.66-48.91]
Skin color (white/black or mixed)	2.49 [0.98-6.35]
**Model 5. BMI + age + smoking habits + skin color + schooling**	
BMI (≥ 25 kg/m^2^)	10.97 [4.27-28.24]
Age (years)	
(30-42)	3.43 [0.94-12.50]
(43-59)	4.10 [0.95-17.80]
(60-88)	14.98 [2.40-93.50]
Skin color (white/black or mixed)	2.63 [1.00-6.88]
Schooling (1-14 years)	1.35 [0.40-4.60]

*OR = odds ratio; 95% CI = 95% confidence interval, BMI = body mass index.*

**Table 5.** Adjusted values for odds ratios and 95% confidence intervals relating to regression analysis models for metabolic syndrome determined for the whole study population in Virgem das Graças, Brazil

Model	OR (95% CI)
**Model 1. BMI**	
BMI (≥ 25 kg/m^2^)	17.88 [8.63-37.05]
**Model 2. BMI + age**	
BMI (≥ 25 kg/m^2^)	23.38 [10.15-53.89]
Age (years)	
(30-42)	2.95 [1.09-8.00]
(43-59)	4.28 [1.43-12.75]
(60-88)	13.24 [3.40-51.43]
**Model 3. BMI + age + marital status**	
BMI (≥ 25 kg/m^2^)	23.18 [10.05-53.43]
Age (years)	
(30-42)	2.82 [1.02-7.85]
(43-59)	3.95 [1.26-12.43]
(60-88)	11.41 [2.48-52.45]
Marital status	
(married)	1.18 [0.34-4.03]
(living with partner)	1.29 [0.27-6.15]
(other)	1.47 [0.24-9.05]
**Model 4. BMI + age + smoking habits**	
BMI (≥ 25 kg/m^2^)	20.84 [8.85-49.09]
Age (years)	
(30-42)	3.38 [1.22-9.36]
(43-59)	5.89 [1.82-19.04]
(60-88)	20.75 [4.98-86.47]
Smoking habits	
(smoker)	1.06 [0.26-4.36]
(former smoker)	0.35 [0.11-1.20]
**Model 5. BMI + age + schooling**	
BMI (≥ 25 kg/m^2^)	21.14 [8.43-50.01]
Age (years)	
(30-42)	3.15 [1.08-9.18]
(43-59)	5.18 [1.38-19.41]
(60-88)	17.58 [3.45-49.51]
Schooling (1-14 years)	1.22 [0.46-3.23]

*OR = odds ratio; 95% CI = 95% confidence interval, BMI = body mass index.*

## DISCUSSION

One important result revealed by the present study was the large and significant difference in the prevalence of MS between men (7.7%) and women (33.6%) in the rural community of Virgem das Graças. This difference may be attributed to the high frequency of abdominal obesity and low concentrations of HDL-C among the women of this community. These findings corroborate the hypothesis that metabolic disorders are more prevalent in females than males, and that high body weight, large waist circumference and low HDL-C are the main contributing factors.^18^ Such differences between the sexes have also been observed in the Canary Islands population.^19^

The age-standardized prevalence of MS in Virgem das Graças was also very high, at 19% (95% CI: 13.7 – 24.3). The frequency of MS increased with age for both sexes, such that approximately 50% of the female population above 60 years of age manifested the syndrome. In contrast, the frequency of MS among men of this age group was only 14.3%. Previous studies in Brazil have focused on the frequency of the individual components of MS. For this reason, the level of occurrence of MS within the whole Brazilian population and the factors that determine its prevalence are still unclear.^20–22^

A further important result deriving from the present study was the strong association detected between MS and overweight, particularly when the whole population was considered. These variables remained significantly associated following adjustment for a number of covariables including age, skin color, marital status, smoking habits and schooling. Recent research in a rural area of Korea^23^ showed that the prevalence of MS was higher despite the low prevalence of obesity or abdominal obesity. However, this was not confirmed in the present study, in which the prevalences of the latter factors were 6.8% and 26.7%, respectively. Longitudinal analyses have demonstrated that BMI plays a central role in the development and identification of MS, and overweight is an important predictor of the syndrome.^24^

It is important to consider the type of criteria used for characterizing MS. One definition has been proposed by the NCEP,^13^ and this was the one used in the present study. The other definition has been adopted by the World Health Organization (WHO).^25^ The latter includes insulin resistance and measurements of micro-albuminuria to detect vascular damage. The two definitions appear to have good agreement; their concordance has been measured as 86.2. In a study involving an American adult population, 23.9% of the individuals were affected by MS according to the NCEP criteria, and 25.1% according to the WHO criteria^26^. These criteria were also valid in a cohort study, as they identify the individuals with a five to nine-fold increased likelihood of diabetes during a follow-up population-based study among middle-age men.^27^

The prevalence of MS varies considerably worldwide. For example, the frequency of MS in a sample of the Chinese population was recorded as 9.8% for men and 17.8% for women.^28^ However, the prevalence of overweight individuals in the same population was much higher; 26.9% for men and 31.1% for women. In a rural area of South Korea, MS was found to affect 29.4% of the adult population above 40 years of age,^23^ and similar values were established in Mexico, where 26.6% of the population studied exhibited the syndrome.^29^

MS is a multiple disorder associated with cardiovascular diseases and is currently perceived as a serious public health problem by national and international authorities worldwide. Within this context, MS is considered to be a pre-diabetic state leading to type II diabetes mellitus.^3^ The extent of the problem is illustrated in the United States population, indicating that 22% of adults presented MS, a frequency that increased to 42% for individuals in the age range of 60 to 69 years of age. The prevalence among African-American women was reported to be even higher still.^26,30^

It is evident that substantial socioeconomic and demographic changes have occurred in the Brazilian population over the past decades and the transition from a rural to an urban lifestyle has been associated with a deterioration of the metabolic profile because of adverse changes in lifestyle habits. Although metabolic syndrome has been a feature of urban westernized lifestyle, risk factors for cardiovascular diseases are now emerging as a major public health problem in developing societies, including rural areas, as demonstrated in this study. To our knowledge, the prevalence of metabolic syndrome has never been studied in a Brazilian rural population and, for this reason, it is difficult to compare our results within the Brazilian context.

The main limitation of the present study, which might restrict the external validity of the analyses performed, derives from the relative small sample population studied. While the number of subjects considered was representative of the adult population of Virgem das Graças, the sample was deficient in young adults who had undergone schooling for a period greater than five years. This is a common difficulty encountered in studies performed in rural areas, since there is a general tendency for young people to migrate to urban centers in search of better education and/or work. In order to overcome this difficulty, the frequency of MS in the whole study population was adjusted according to the age profile, in comparison with a standard population from a rural area in Minas Gerais.^17^

## CONCLUSIONS

The present study demonstrated that MS is a serious problem among this poor rural community, affecting primarily women and individuals over 60 years of age. The prevalence of MS in these two groups warrants further investigation using a larger rural sample population, since the prevention and treatment of this condition is of major public concern and urgently requires the application of appropriate policies and considerable investment.
